# The Effects of Substituting BHT with a Microencapsulated Basil (Ocimum campechianum Mill.) Extract on Refrigerated Beef Burger Preservation

**DOI:** 10.3390/foods15071221

**Published:** 2026-04-03

**Authors:** Rafaela Borges do Vale, Aurianna Coelho Barros, Izanildo de Jesus Maciel, Shaenna Karlla de Sousa Melo, Talita Raquel Rodrigues da Silva, Jônatas José da Silva, José Anderson da Silva, Juliana dos Santos Lima, Maria Gracileide de Alencar, Mateus Matiuzzi da Costa, Rafael Torres de Souza Rodrigues, Ana Julia de Brito Araujo Carvalho, Marcos dos Santos Lima, Begoña Panea Doblado, Francisco Allan Leandro de Carvalho

**Affiliations:** 1 Campus Ciências Agrárias, Universidade Federal do Vale do São Francisco, Rodovia BR 407, 12 Lote 543-Projeto de Irrigação Nilo Coelho-S/N C1 CEP, Petrolina 56300-000, PE, Brazil; rafaelaborgesdovale@gmail.com (R.B.d.V.); aurianna1604@gmail.com (A.C.B.); izanildomaciel@hotmail.com (I.d.J.M.); shaennamelo@outlook.com (S.K.d.S.M.); talitazootec@gmail.com (T.R.R.d.S.); reservajonatas1996@gmail.com (J.J.d.S.); joseandersonn2007@gmail.com (J.A.d.S.); juliana.santoslima@discente.univasf.edu.br (J.d.S.L.); gracileide_alencar@hotmail.com (M.G.d.A.); mateus.matiuzzi@gmail.com (M.M.d.C.); rafaeltsrodrigues@gmail.com (R.T.d.S.R.); francisco.allan@univasf.edu.br (F.A.L.d.C.); 2 Instituto Federal do Sertão Pernambucano, R. Maria Luzia de Araújo Gomes Cabral, 791–João de Deus, Petrolina 56309-680, PE, Brazil; ana.julia@ifsertao-pe.edu.br (A.J.d.B.A.C.); marcos.santos@ifsertao-pe.edu.br (M.d.S.L.); 3 Centro de Investigación y Tecnología Agroalimentaria de Aragón, Departamento de Ciencia Animal, Avda. Montañana 930, 50059 Zaragoza, Spain; 4 Instituto Agroalimentario de Aragón, IA2-CITA, Avda. Miguel Servet, 177, 50013 Zaragoza, Spain

**Keywords:** bioactive compounds, preservation, lipid oxidation, meat product

## Abstract

The antioxidant efficiency of basil extract (*Ocimum campechianum Mill*) was evaluated in chilled (4 °C) beef hamburgers. Six treatments were prepared: control (CON), synthetic antioxidant (BHT), basil extract at 0.01% and 0.02% (BE1 and BE2, respectively), and microencapsulated basil extract at 0.01% and 0.02% (ME1 and ME2, respectively). The extracts were analyzed for their antimicrobial activity, antioxidant capacity, and phenolic compound profile. Burger physicochemical properties, lipid oxidation (peroxide value and TBARSs), colour, pH, texture, and sensory acceptance were analyzed during storage. Treatment affected both DPPH and FRAP values. A total of 28 phenolic compounds were identified. Treatments with basil extract helped control pH and reduced peroxide formation compared with the control, while colour and technological parameters were not significantly affected. TBARS values remained low across treatments. Sensory evaluation indicated lower acceptability for BE2 and ME2, although no significant differences in overall preference were observed. Consequently, basil extract, whether microencapsulated or not, can be utilized as a substitute for synthetic antioxidants in refrigerated beef hamburgers.

## 1. Introduction

The consumption of processed meat products has grown significantly in recent years, both globally and in Brazil, due to their convenience, affordability, and versatility [1]. Among them, burgers are a popular choice due to their ease of preparation and universal appeal. Nonetheless, meat products are highly susceptible to spoilage due to their chemical composition and high water content. These factors permit the growth and action of microorganisms, which in turn cause sensory changes, including alterations in colour, flavour, texture and aroma as well as nutritional changes related to the oxidation of lipids and proteins [2]. These deterioration processes are especially significant in grinded meat, such as burgers, since this meat is more exposed to factors stimulating lipid oxidation and the formation of toxic compounds [3]. Therefore, with the aim of preventing or delaying oxidative reactions in meat products, synthetic antioxidants such as butylated hydroxytoluene (BHT) have been widely used by the food industry. However, Brazilian Resolution RDC No. 272 (2019) [4] banned the use of BHT in food due to possible health risks associated with continuous and prolonged consumption of these compounds [5]. Moreover, consumers are rejecting synthetic ingredients and searching for more natural alternatives. Consequently, it is necessary to explore alternative solutions to ensure the safety and shelf-life of meat products. A substantial body of literature exists on the utilization of plant extracts and essential oils as natural antioxidants, including aromatic plants, fruits, leaves, seeds, and spices [6]. Among them, it has been reported that ethanolic extracts from basil leaves have significant antioxidant potential and antibacterial activity against Gram-positive and Gram-negative bacteria [7,8]. This activity has been attributed to phenolic acids, flavonoids, gallic acid, and aromatic compounds present in their essential oils, including eugenol, chavicol, linalool, and α-terpineol [9,10]. Therefore, basil has been used as an ingredient in the production of films and coatings [11,12,13,14], combined with plastics in absorbent pads [15,16] and used as an ingredient in animals’ diets [17,18,19]. Concerning meat and meat products, gum from basil seeds has been used as a fat replacer in some products, mainly chicken products [11,20], whereas oils have been combined with whey protein isolate-almond gum as an encapsulation agent for chicken meat [21]. Surprisingly, the direct use of basil seeds as an ingredient in a meat product is still scarcely reported and most of the experiments are carried out with chicken or pork products [22,23,24,25], while few studies are focused on beef products [26].

On the other hand, some of the bioactive basil compounds are sensitive to heat or light and may lose their bioactivity when used as ingredients in a processed product. To address this challenge, microencapsulation is emerging as a viable solution to preserve the functional integrity of bioactive compounds [27,28] but the encapsulation process has not been fully explored for basil extracts. In fact, to the authors’ knowledge, this is the first study combining the encapsulation process of a basil extract and it use as an ingredient in a beef burger. The objective of this study was to ascertain whether the incorporation of a natural extract of basil, encapsulated or not, would result in an extension to the preservation of beef burgers.

## 2. Materials and Methods

### 2.1. Experiment Location and Ethical Aspects

The study was conducted at the Laboratory of Animal Product Technology (Meat and Fish), the Laboratory of Bromatology and Animal Nutrition, and the Laboratory of Animal Microbiology and Immunology, all belonging to the Agricultural Sciences Campus of the Federal University of Vale do São Francisco (UNIVASF), located in Petrolina, Pernambuco, Brazil. The research was submitted to Plataforma Brasil and approved by the UNIVASF Research Ethics Committee (CEP) (Protocol number: 77949224.9.0000.0282).

### 2.2. Obtaining and Characterizing Microencapsulated Basil Extract

Basil leaves from mature plants in their first flowering cycle (at 60 days) were collected in the municipalities of São Gabriel and Cotegipe, Bahia. To obtain the extract, the leaves (1198.70 g) were first dried in a forced-air oven (Model NT 534, Novatecnica, Piracicaba, São Paulo, Brazil) at 45 °C, processed in a knife mill (Wiley, Marconi, MA-580, Piracicaba—São Paulo, Brazil) and passed through a 28-mesh stainless steel sieve (Prolab, São Paulo/SP, Brazil). The ground material was placed in a percolator and washed repeatedly with ethanol (99.3%) (CAS: 64-17-5), and the extracted liquid was collected every 72 h. This solution was filtered through qualitative filter paper and concentrated in a rotary evaporator (IKA RV 3, IKA Brasil, Campinas, São Paulo, Brazil) at 50 °C. The material was then dried in a forced-air circulation oven (Model NT 534, Novatecnica, Piracicaba, São Paulo, Brazil) at 45 °C for 72 h. The final yield of the extract was 5.72 g/100 g of leaves. After obtaining the extract, it was stored in a sterile amber glass container, sealed and stored under refrigeration (4 °C) for further analysis.

One aliquot of this extract was stored as free extract (referred to as free basil extract, BE), while another aliquot was microencapsulated (referred to as microencapsulated basil extract, ME).

Spray dryer microencapsulation was performed at the Experimental Food Laboratory (LEA) of the Federal Institute of Education, Science, and Technology of Sertão Pernambucano (IF Sertão, PE), following the methodologies described by Vongsak et al. [29] and Tsali and Goula [30] with modifications made by Amorim et al. [31]. The microencapsulation process was performed by diluting 92.77 g of the sample in a 1:10 ratio in 50% ethanol, and 15% maltodextrin was added. Spray drying to obtain the microencapsulated powders was performed in a Spray Dryer (model MSDi 1.0, Labmaq, Ribeirão Preto, São Paulo, Brazil) with an air-drying temperature of 140 °C. This drying temperature was established after investigating the impact of drying temperature on quality parameters (including antioxidant activity). An air flow of 25 m/s was used, with an average volumetric flow rate of 200 mL/h (20% of the equipment’s capacity), a 1.0 mm spray nozzle, and a drying air flow of 35 L/min.

### 2.3. Antioxidant Capacity of the BEs and MEs

#### 2.3.1. Reagents

For the DPPH method, methanol (CAS: 67-56-1) from Dinâmica Química Contemporânea LTDA was used, as well as Trolox (CAS: 53188-07-1) and DPPH (CAS: 1898-66-4), both acquired from Sigma-Aldrich. In the determination of the antioxidant capacity by the FRAP method, the reagents used were: TPTZ Tripyryltriazine (CAS: 3682-35-7) from SIGMA-ALDRICH, hydrochloric acid (CAS:1310-73-2), ferric chloride (CAS: 10025-77-1), acetate buffer (CAS: 126-96-5), ferrous sulfate (CAS: 7782-63-0) and glacial acetic acid. For the total phenolics, the gallic acid (CAS: 5995-86-8), methanol (CAS: 67-56-1) and sodium carbonate (CAS: 497-19-8) were all acquired from the company Dinâmica Química Contemporânea LTDA (Indaiatuba, São Paulo, Brazil).

#### 2.3.2. Antioxidant Activity by Scavenging the DPPH (2,2-diphenyl-1-picrylhydrazyl) Radical

The DPPH method was performed according to the procedure described by Brand-Williams et al. [32] and Zhou et al. [33]. Briefly, 100 μL of previously prepared samples of the extract and microencapsulated basil extract was added to 3.9 mL of DPPH solution (60 μM in methanol). After incubation in a water bath (Nova Instruments, NI 1248, Piracicaba, São Paulo, Brazil) at 37 °C for 10 min, absorbance readings were taken at 515 nm in a KASVI spectrophotometer (K37-UV/VIS, LumiLabor Itaquera, São Paulo, Brazil). The DPPH scavenging activity of the extracts was determined using Trolox as a standard, and the results were expressed in mg Trolox/g of sample.

#### 2.3.3. Ferric Reducing Antioxidant Power (FRAP)

Ferric reducing antioxidant power was determined using the method described by Benzie and Strain [34]. The FRAP reagent was prepared by mixing 25 mL of acetate buffer solution (300 mM; pH 3.6), 2.5 mL of TPTZ solution (10 mM TPTZ in 40 mM HCl) and 2.5 mL of FeCl_3_ (20 mM) in aqueous solution. The three solutions were mixed in a 10:1:1 (v:v:v) ratio. Next, 900 μL of freshly prepared FRAP reagent was mixed with 30 μL of appropriately diluted samples and 90 μL of distilled water. The mixture was kept at 37 °C in a water bath (Nova Instruments, NI 1248, Piracicaba, São Paulo, Brazil) for 20 min, and the absorbance was measured at 593 nm in a KASVI spectrophotometer (K37-UV/VIS, LumiLabor Itaquera, São Paulo, Brazil). The FRAP value was expressed as μmol Fe^+2^/100 g of sample based on a calibration curve prepared using FeSO_4_ as a standard.

#### 2.3.4. Total Phenolic Content

The concentration of total phenolics was determined by the Folin–Ciocalteu spectrophotometric method [35]. Thus, 50 μL of the sample, 3.95 mL of distilled water and 250 μL of the Folin–Ciocalteu reagent were added to a test tube. After 3 to 8 min, 750 μL of saturated 20% sodium carbonate solution was added, remaining at rest for 2 h. Next, the absorbance at 765 nm was read in a 10 mm glass cuvette (path length) using a UV–Visible spectrophotometer (model UV 2000A, Instrutherm, São Paulo, Brazil) after zeroing the instrument with a blank solution. The results were expressed in mg/Kg of equivalent to gallic acid, by comparison with a calibration curve constructed at concentrations of 0, 25, 50, 100, 250, 350 and 500 mg/L of gallic acid.

#### 2.3.5. Analysis of the Profile of Individual Phenolic Compounds by HPLC-DAD

The determination of individual phenolic compounds was performed following the method validated by dos Santos Lima et al. [36]. The analysis was performed using a liquid chromatograph (Agilent 1260 model, Santa Clara, CA, USA), equipped with a quaternary pump (model G1311C) and gas, automatic sample injector (model G1329B), and thermostatic oven, and a gas chromatograph detector (model G1329B, Santa Clara, CA, USA), equipped with a quaternary pump (model G1311C, Santa Clara, CA, USA) and gas, automatic sample injector (model G1329B, Santa Clara, CA, USA), thermostatic oven for columns (model G1316A, Santa Clara, CA, USA), and diode array detector—DAD (model G1315D, Santa Clara, CA, USA). Separation was performed using a Zorbax Eclipse Plus RRHT RP-C18 ultra-fast resolution column (50 × 4.6 mm; with 1.8 μm particles, Santa Clara, CA, USA), maintained at 40 °C, with a sample injection volume of 10 μL. The mobile phase flow rate was 1.0 mL/min, with phase A being an acidified solution of 0.5% (*v*/*v*) orthophosphoric acid and phase B being methanol acidified with 0.5% (*v*/*v*) orthophosphoric acid, following the gradient: 0 min: 0% B; 2 min: 10% B; 13 min: 26% B; 19 min: 50% B; 21 min: 80% B; 21.1–23.1 min: 100% B; 23.2 min: 0% B (3 min post-run). The detection of phenolic compounds was performed at wavelengths of 220, 280, 320, 360, and 520 nm using a DAD. The results obtained were processed using OpenLAB CDS ChemStation software (Version 2.8, Agilent Technologies, Santa Clara, CA, USA).

### 2.4. Antimicrobial Activity of the BEs and MEs

The Minimum Inhibitory Concentration (MIC) and the Minimum Bactericidal Concentration (MBC) of both BEs and MEs were determined using the following reference strains from the American Type Culture Collection, available at the Laboratory of Animal Microbiology and Immunology of the Federal University of Vale do São Francisco: *Staphylococcus aureus*—ATCC 29213, *Salmonella enteritidis*—ATCC 13076, *Escherichia coli*—ATCC 10523, *Escherichia coli*—ATCC 10536, *Klebsiella pneumoniae*—ATCC 13883, *Staphylococcus aureus*—ATCC 33591, and *Staphylococcus aureus*—ATCC 25923. The bacterial inoculum was prepared according to protocol M07-A9 of the Clinical and Laboratory Standards Institute [17]. In brief, the procedure was as follows: 25000 μL solutions of free basil extract (BE) and microencapsulated basil extract (ME) were diluted in 10% dimethyl sulfoxide P.A. (DMSO, Neon Comercial Ltd.a, Suzano, Brazil). Subsequently, 10 μL of bacterial suspension at 1.5 × 10^6^ CFU/mL was added, and the microplates were incubated for 24 h at 37 °C. Next, 30 μL of 2,3,5-triphenyl tetrazolium chloride (CTT, Dinâmica, Indaiatuba, Brazil) was added to each well and incubated for 1 h. The lowest concentration of the test substance capable of inhibiting bacterial growth was considered the MIC. Subsequently, a 10 μL aliquot from each well was inoculated in MH agar (Kasvi, Weissópolis Pinhais, Paraná, Brazil) (CAS: 9002-18-0) with the replicator support and incubated (Cislab, Guarulhos, São Paulo, Brazil) at 37 °C for 24 h. The MBC was considered the lowest concentration of the test substance capable of causing bacterial death. The tests were performed in triplicate on three independent days.

### 2.5. Preparation and Sampling of Burgers

To manufacture burgers, 88.49% lean beef meat (moisture: 70.56%; ash: 0.84%; protein: 21.34%; and lipids: 6.85%) and 10% pork fat were used, both purchased from local markets. Each treatment used 1500 g of beef and 150 g of pork fat, which were ground in a meat grinder (model BM 20 NR PR 1.25 hp 220 V, Bermar, São José do Rio Preto, São Paulo, Brazil) using 8 mm discs for the beef and 6 mm discs for the pork fat and mixed manually with 24.75 g (1.5%) of salt until completely homogenized. Then, the BHT or the corresponding extracts were added, depending on the treatment. Six treatments, with two batches per treatment, were prepared:

- Control (CON) without antioxidant addition;
- BHT, with 0.015 g of BHT, corresponding to 0.01% of the percentage of fat, which is the concentration established by Brazilian legislation for burger production;
- BE1 with 0.015 g (0.01%) of free basil extract;
- BE2 with 0.030 g (0.02%) of free basil extract;
- ME1 with 0.015 g (0.01%) of microencapsulated basil extract;
- ME2 with 0.030 g (0.02%) of microencapsulated basil extract.

For each treatment, six hamburgers were prepared for analysis on day 0, three for analysis on day 7, another three for analysis on day 14, and six for analysis on day 21, for a total of 18 hamburgers per treatment. Two batches were made for each treatment, resulting in a total of 36 hamburgers per treatment.

**Figure 1:**
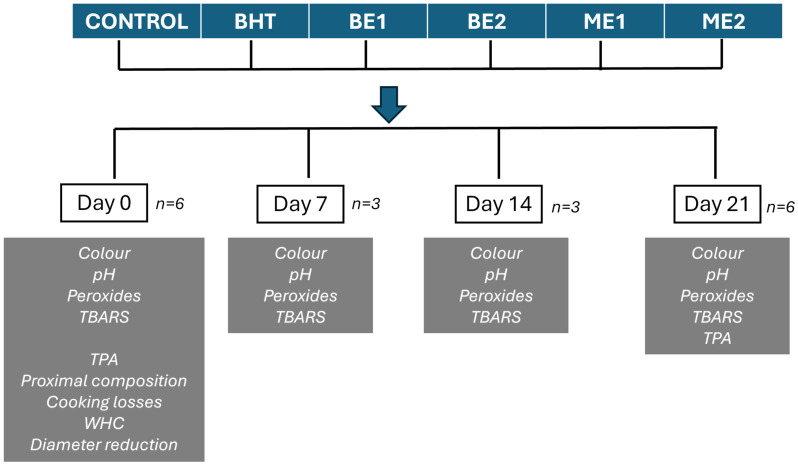
Scheme of experimental design.

All the burgers were vacuum-packed in polypropylene bags and stored under refrigeration (4 °C). The centesimal composition, water-holding capacity, water loss during cooking, diameter reduction, and sensory analysis were assessed only on elaboration day (day 0). The parameters of pH, colour, and oxidative stability (peroxides and TBARS) were analyzed on days 0, 7, 14, and 21, and texture (TPA) was analyzed on days 0 and 21. A scheme of the design is shown in Figure 1.

### 2.6. Meat Analysis

#### 2.6.1. Centesimal Composition

The centesimal composition of fresh meat and burgers was assessed on day 0 in triplicate, following the methods described by the Association of Official Analytical Chemists (AOAC, 2016) [37]. Moisture was determined by weighing 1 g of the sample until a constant weight was reached in a drying oven at 105 °C (model S80ar Biopar, Camargo Industrial, São Carlos, São Paulo, Brazil) (method 967.03). Mineral matter (sample from moisture determination) was determined in a muffle furnace at 600 °C (GP Científica, Tatuapé, São Paulo, Brazil) for 5 h (method 942.05). Crude protein was determined by the Kjeldahl method (model MA 036, Marocni, Piracicaba, São Paulo, Brazil) (method 981.10) by weighing 0.1 g of the sample. For the lipid content, 1 g of the sample was weighed and determined in an extractor (ANKOM TX-10, ANKOM Technology—Macedon, NY, USA) according to the methodology proposed by the AOCS [38].

#### 2.6.2. Water-Holding Capacity (WHC)

Water-holding capacity (WHC) was obtained using the filter paper pressure method [39,40]. Samples of 0.5 g of burgers were placed on filter paper with an area of 10 × 10 cm^2^ (Whatman No. 1 paper) between two Plexiglas plates. The assembly was pressed with a weight of 5 kg for 5 min and the samples were then weighed again. WHC was calculated based on the difference between the initial and final weight, expressed as a percentage:

WHC (%) = (initial weight – final weight) × 100

#### 2.6.3. Cooking Losses (CLs)

To determine cooking losses, the burgers were weighed on an analytical scale (Model JF2204AT—BIOSCALE, Toledo, Paraná, Brazil) and grilled on a grill (Model Premium G-03, MONDIAL, Cruz das Almas, Bahia, Brazil) until they reached 72 °C, controlled by a digital skewer thermometer (Netlab, Tatuapé, São Paulo, Brazil) pointed at the geometric centre of the burger [41]. Subsequently, the burgers were cooled at a room temperature of 20 ± 2 °C and weighed again. The PPC value was calculated based on the difference between the initial and final weight, expressed as a percentage:

$$CL\left(\%\right)=\frac{Initial\;weight-Final\;weight}{Initial\;weight}\times 100$$

#### 2.6.4. Diameter Reduction

The diameter reduction (DR) was measured according to the methodology described by Fontan, Reboucas, Verissimo, Machado, Fontan and Bonomo [41]. For this purpose, the diameter of the raw and cooked burgers, cooked as explained above, were measured using a digital calliper (Model 316119-MTX, São Paulo, São Paulo, Brazil). The DR was calculated based on the difference between the initial and final diameter, expressed as a percentage:

$$DR\left(\%\right)=\frac{Diameter\;of\;raw\;sample-Diameter\;of\;cooked\;sample}{Diameter\;of\;raw\;sample}\times 100$$

#### 2.6.5. pH and Instrumental Colour

The pH analysis of the burgers was performed in triplicate using a Hanna digital insertion pH meter (TAO4410386, Hanna Instruments, Nușfalău, Romania), previously calibrated with two standards (pH 4.0 and 7.0), inserted into the centre of the samples.

Colour was measured using a 3 nh NR110 colorimeter (Shenzhen, China), at 0% UV, D65 illuminant, an observation angle of 10º and measurement area of 8 mm, with zero and white calibration, in the CIE L*a*b* 1986 space. The burgers were removed from their packaging, placed on glass plates, and measurements were taken after 15 min of exposure to an ambient atmosphere (20 ± 2 °C) at three different points. The luminosity (L*), redness (a*) and yellowing (b*) indices were recorded. The average of the three readings was used for statistical analysis. Chroma [$C_{ab}^{*}=\sqrt{{\left(a^{*}\right)}^{2}+{\left(b^{*}\right)}^{2}}$] and hue [$h_{ab}=\tan^{-1}\left(\frac{b^{*}}{a^{*}}\right)\cdot \frac{180}{\pi }$] were calculated [42].

#### 2.6.6. Texture Profile Analysis (TPA)

Samples used to determine cooking losses were thereafter used for texture profile analysis. Texture profile analysis (TPA) was performed using a TA-TX2 Expression Express texturometer (Stable Micro Systems, version 1.1.12.0 Surrey, UK) connected to a computer. The burgers from the PPC analysis were cut into 2 cm × 2 cm samples and subjected to analysis with a double compression cycle performed up to 50% compression of the height of the original portion with a 45 mm diameter aluminum probe. The texture profile parameters hardness (N), chewiness (N/mm), elasticity (mm), and cohesiveness were determined according to Bourne [43].

#### 2.6.7. Lipid Oxidation: Peroxide Index and TBARS

For peroxide index and TBARS analysis, the following reagents were used: acetic acid (CAS: 64-19-7), chloroform (CAS: 67-66-3), saturated potassium iodide solution (CAS: 7681-11-0), sodium thiosulfate solution (CAS: 10102-17-7), starch solution (CAS: 9005-84-9), trichloroacetic acid (TCA) (CAS: 76-03-9), M 2-thiobarbituric acid (CAS: 504-17-6), and tetraethoxypropane (TEP) (CAS: 122-31-6). All reagents were purchased from Êxodo Científica, Sumaré (São Paulo, Brazil), except TEP, which was purchased from Sigma-Aldrich Brasil Ltda (Barueri, São Paulo, Brazil).

The peroxide index was determined according to the methods described by the AOCS [44] with adaptations. We weighed 2.5 g of the sample in a 125 mL Erlenmeyer flask, added 15 mL of a 3:2 acetic acid–chloroform solution and shook the mixture until the sample was thoroughly homogenized. Then, 0.25 mL of saturated potassium iodide (KI) solution was added, after which the mixture was left to stand in the dark for exactly one minute. Next, 15 mL of water was added for subsequent titration with 0.01 N sodium thiosulfate solution, with constant stirring, until the yellow colour faded. Finally, 0.25 mL of starch solution was added as an indicator, after which titration was continued until the blue colour disappeared completely. The peroxide index was expressed in mEq/kg of sample.

The measurement of 2-thiobarbituric acid reactive substances (TBARS) was determined according to the method proposed by Vyncke [45], with adaptations. A burger sample (2 g) was dispersed in 10 mL trichloroacetic acid (TCA) at 7.5% and homogenized in Ultra-Turrax (Ika T25 basic, Staufen, Germany) for 30 s. The homogenate was kept at −18 °C for 10 min. The supernatant was filtered through filter paper (Whatman No. 1). In the filtered solution, 5 mL of 0.02 M 2-thiobarbituric acid (TBA) solution was added and then incubated in a water bath at 98 °C for 40 min. After the water bath, the samples were placed in an ultrasonic bath (7 LAB, SSBU) for 5 min. The absorbance was measured in a KASVI spectrophotometer (K37-UV/VIS, LumiLabor, Itaquera, São Paulo, Brazil) at 532 nm. The TBARS value was calculated from a standard curve of malonaldehyde with tetraethoxypropane (TEP) and expressed in mg of malonaldehyde per kg of sample (mg MDA/kg).

#### 2.6.8. Sensory Analysis and Purchase Intention

The project was approved by the UNIVASF Research Ethics Committee (Protocol number: 77949224.9.0000.0282). Prior to the sensory test, the safety of the samples was verified by a microbiological analysis including counts of *Escherichia coli*, *Salmonella* spp., and coagulase-positive staphylococci microorganisms [46]. All the counts were within the limits established by law, as described in the normative instruction No. 313 of September 4, 2024 [47].

The test was carried out at the facilities of the university (UNIVASF), using standardized individual booths. Burgers were grilled as described above. Then, they were cut, identified (3 random digits), and kept warm until testing. An affective acceptance test was conducted with a convenience sample of 105 untrained individuals, who evaluated the product in terms of colour, aroma, flavour, texture, and overall quality, using a structured 9-point hedonic scale ranging from 9 = “I liked it very much” to 1 = “disliked very much,” and a test of purchase intention was conducted for the product using a five-point scale ranging from 5 = “I would definitely buy it” to 1 = “I would definitely not buy it.”

### 2.7. Statistical Analysis

A completely randomized experimental design with six treatments and 4 sampling times was used. Initially, the data were tested for normality and homogeneity of variance (Shapiro–Wilk). The PROC MIXED package of the Statistical Analysis System (SAS, Version 9.1, 2009) was used. The data were then submitted to two-way analysis of variance (ANOVA) with treatment and storage time as fixed effects. One-way analysis of variance was applied to the variables cooking losses, water retention capacity, diameter reduction, and proximal composition, evaluated only on day 0, followed by Tukey’s test when ANOVA was significant (*p* < 0.05). Sensory data were analyzed using the sensory package of XLSTAT V2025.1.3. First, a liking analysis was performed, centring and scaling the consumers’ scores to reduce variability due to different uses of the scale. Next, a cluster analysis was performed to group consumers. Subsequently, an ANOVA was performed to detect differences between clusters. For purchase intention, the percentages of purchase intention were calculated for each category and product, both with the general data and within each cluster, and the differences between clusters were studied using a chi-square test.

## 3. Results and Discussion

### 3.1. Antioxidant Capacity of Basil Extracts

The antioxidant activity of the extracts and butylated hydroxytoluene (BHT) is presented in Table 1. Regarding DPPH (2,2-diphenyl-1-picrylhydrazyl) radical scavenging, basil extracts (BEs) exhibited lower antioxidant activity than microencapsulated extracts (MEs) and BHT, whereas no significant differences were observed between ME and BHT. The antioxidant activity of a plant extract is dependent on the cultivar and the methodology of the extraction process [10]. Working on beef sausages with basil extracts, Macari, Stürza, Lung, Soran, Opriş, Balan, Ghendov-Moșanu, Cristian Vodnar and Cojocari [26] reported values of 490.6 mg TE/g. In contrast, Vlaicu et al. [48] described values of 4985.3 mg TE/g in a basil ethanolic extract, whereas Kaczmarek and Muzolf-Panek [24] reported values of 33.7 mg TE/g in a water:ethanol extract. Furthermore, a review conducted by Sharifi-Rad et al. [49] indicated that basil exhibits DPPH IC_50_ values ranging from 0.00022 to 0.02049 mg/mL, depending on the solvent and extraction method, indicating moderate antioxidant activity of basil when compared with other plants.

For the ferric reducing antioxidant power (FRAP) assay, basil extracts (BEs) exhibited the highest values, followed by microencapsulated extracts (MEs) and BHT. The FRAP activity of BE was nearly tenfold higher than that of BHT, highlighting the strong reducing capacity of the non-encapsulated extract. These findings are consistent with those reported in previous studies. For example, Naher et al. [50] reported FRAP values of 836–2423 µmol Fe^+2^/g extract in ethanolic basil extracts, depending on the ethanol concentration. In a study conducted with chicken nuggets, Nadeem, Akhtar, Ismail, Qamar, Sestili, Saeed, Azeem and Esatbeyoglu [25] reported values of 23.4 µmol Fe^+2^/g.

The antioxidant capacity of a sample can vary depending on the assay employed due to the distinct chemical mechanisms involved [51]. Free extracts provide immediate accessibility of antioxidants to the assay reagents, allowing for rapid reduction of Fe^3+^ in the FRAP assay and resulting in elevated reductive signals [52]. In contrast, volatilization, aggregation, or suboptimal dispersion may slightly limit their apparent DPPH radical scavenging [51,53]. Encapsulation in maltodextrin, however, stabilizes labile phenolic and volatile compounds, protects them from thermal and oxidative degradation, and improves their dispersion in polar or mixed solvents, thereby enhancing apparent DPPH activity [53]. Simultaneously, the polymeric matrix can physically retain antioxidants and slow their release, limiting contact with the aqueous ferric reagent in the FRAP assay and producing lower measured values [52,54]. Thus, encapsulation conserves and disperses antioxidants effectively in the DPPH environment but restricts immediate interaction with the FRAP reagent, while free extracts, fully exposed, exhibit maximal FRAP response [54].

Therefore, overall, basil—whether encapsulated or not—demonstrates equal or superior antioxidant capacity compared with BHT, highlighting its potential as a natural alternative to synthetic antioxidants.

### 3.2. Profile of Individual Phenolic Compounds

Table 2 shows the profile of phenolic compounds in basil extract (BE) and microencapsulated basil extract (ME). A total of 28 antioxidant compounds were quantified, comprising 10 phenolic acids, 2 stilbenes, 1 phenolic aldehyde, 4 isoflavones, 3 flavan-3-ols, 3 proanthocyanidins, and 5 flavonols. Among these, naringenin was the most abundant, with an average concentration of 294.72 mg/kg. The total phenolic content was higher in BE (631.95 mg/kg) than in ME (469.54 mg/kg); however, only four individual compounds—fumaric acid, epigallocatechin gallate, quercetin 3-glycoside, and rutin—exhibited statistically significant differences (*p* < 0.05) between the two extract forms. The concentrations of epigallocatechin gallate and quercetin 3-glycoside were higher in BE than in ME, whereas rutin was more abundant in ME. Notably, fumaric acid was not detected in ME. It was expected that the overall phenolic content would be lower in the encapsulated extract [55]. Although it has been demonstrated that microencapsulation has the capacity to protect bioactive compounds, particularly through the employment of the spray-drying technique, due to the rapid nature of this method [56], some authors [57] indicated that certain phenolic compounds could undergo degradation throughout the drying process, so they may not be detected in the microencapsulated mixture. The release rate of encapsulated compounds is dependent on the properties and amount of the encapsulated substance as well as on the parameters that regulate the spray-drying procedure [55,58], and several authors indicate that the concentration of total phenolic compounds decreased when the number of drying cycles or drying temperature increased [59,60]. Other factors reported by the literature explaining the reduction in total phenolics during the drying process are the formation of surface cracks or pores, moisture evaporation or moisture sublimation [61].

Romano et al. [62], studying basil leaves and different extraction methods, stated that most abundant compounds were terpenes, followed by alcohols and aldehydes, which disagrees with the current results. Nevertheless, they concluded that acids represented around 12% of TPC, which is closer to our results. In contrast, Majdi, Pereira, Dias, Calhelha, Alves, Rhourri-Frih, Charrouf, Barros, Amaral and Ferreira [10] described values ranged 52.4–91 mg/g for acid content, which represents 86% of total phenolic content. In the same way, Naher, Nilsuwan, Palamae, Hong, Zhang, Osako and Benjakul [50] described that flavonoids accounted for almost 50% of the total polyphenol content (TPC), whereas the current results indicate a percentage of around 80%. All these discrepancies are due to the cultivars and extraction procedures and solvents.

### 3.3. Antimicrobial Capacity of Basil Extracts

Table 3 shows the minimum inhibitory concentration (MIC) and minimum bactericidal concentration (MBC) of BE and ME against the tested bacterial strains. BE exhibited antimicrobial activity against all six tested strains. A concentration of 6.25 mg/mL was effective against the Gram-positive *Staphylococcus aureus*, whereas Gram-negative strains (*Salmonella enteritidis, Escherichia coli*, and *Klebsiella pneumoniae*) required a minimum effective concentration of 12.5 mg/mL. Notably, the minimum bactericidal concentration (MBC) for *Klebsiella pneumoniae* exceeded the detection limit of the method (12.5 mg/mL). For BE, MIC values were equal to MBC values across all tested strains, suggesting a direct bactericidal effect [63]. Regarding ME, bacterial growth was still observed at a concentration of 12.5 mg/mL. This reduced antimicrobial activity is likely attributable to encapsulation, which may delay the release and action of the active compounds [64].

Most studies report that basil extracts exhibit antimicrobial activity, particularly against Gram-positive bacteria, which aligns with the findings of the present study. Sharifi-Rad, Adetunji, Olaniyan, Ojo, Samuel, Temitayo, Roli, Nimota, Oluwabunmi, Adetunji, Sharopov, Cruz-Martins and Contreras [49] reported that basil has antimicrobial activity at concentrations ranging from 50 to 200 mg/mL, depending on the study’s conditions. Macari, Stürza, Lung, Soran, Opriş, Balan, Ghendov-Moșanu, Cristian Vodnar and Cojocari [26] reported MIC values ranging from 1.25 mg/mL to 5 mg/mL and MBC values ranging from 2.5 mg/mL to 10 mg/mL against several strains, including *Listeria*, *Pseudmonas*, *Salmonella* and *E. coli*. Majdi, Pereira, Dias, Calhelha, Alves, Rhourri-Frih, Charrouf, Barros, Amaral and Ferreira [10] described MIC values of 20 mg/L and MBC values above 20 mg/mL against Gram-positive bacteria. Takwa, Caleja, Barreira, Soković, Achour, Barros and Ferreira [27] described a range of MIC and MBC values from 0.15 to 0.45 mg/mL and from 0.30 to 0.60 mg/mL, respectively. Sharafati-Chaleshtori et al. [65] reported values of 1560 μL/mL and 6250 μL/mL for MIC and MBC, respectively, highlighting the effect against *Staphylococcus*. Gaio et al. [66] reported MIC values from 0.25 mg/L to 1 mg/mL and stated that basil did not present activity against *P. aeruginosa*. The primary agents responsible for the antimicrobial activity are terpenes, which disrupt bacterial osmoregulation, alter membrane permeability, and ultimately lead to cell collapse. Nevertheless, the antimicrobial activity of basil is moderate compared with that of other plant extracts. Marmion et al. [67] observed that basil exhibited antimicrobial activity at concentrations exceeding 1% (*v*/*v*), while other plants, such as oregano and thymus, demonstrated activity at concentrations 10 times lower (0.1–0.5%) (*v*/*v*).

### 3.4. Meat Quality

#### 3.4.1. Centesimal Composition and Technological Properties

The results for the centesimal composition and technological properties of the burgers are presented in Table 4. The values for the centesimal composition of the burgers are in accordance with those established by Brazilian legislation [68]. There were significant differences (*p* < 0.05) in the moisture and fat percentages among the treatments. Treatment BE1 had the highest moisture content, differing only from the control (CON). The other treatments with either BE or ME did not differ from the control or the BHT. The fat percentage was higher in the control (CON) treatment, which was only significantly different from BE2, which showed the lowest values. The other treatments showed intermediate values that were not different from the CON or BHT. There were no significant differences between the treatments for either ash or protein content (*p* > 0.05).

Statistically significant differences (*p* < 0.05) were observed among treatments for water-holding capacity (WHC), cooking losses, and diameter reduction. For WHC, no significant differences were found between BHT and CON or between BHT and any basil-added treatment. BE1 tended to show the highest WHC, while ME1 exhibited the lowest. Cooking losses did not differ significantly among the four basil-added treatments or between these and CON, whereas BHT showed smaller losses than CON, BE2, ME1, and ME2 (*p* < 0.05). Regarding diameter reduction, only BE2 differed significantly from CON and BHT, showing the smallest reduction, followed by ME2.

Despite similar protein contents, differences in WHC, cooking losses, and shrinkage were largely influenced by moisture and fat levels. Higher-moisture, lower-fat formulations (e.g., BE1) exhibited enhanced WHC, resulting in smaller cooking losses and dimensional shrinkage, whereas higher-fat treatments (e.g., CON) showed reduced WHC, indicating that fat limits water retention in the matrix [69,70]. The functional effects of basil extracts are attributed to their polyphenol- and terpenoid-rich composition, which interacts with meat proteins to stabilize the network, retain moisture, and reduce fat migration, particularly when encapsulated [20,25,70,71]. These effects occur without altering total protein or ash content, consistent with previous reports [69,70]. Mechanistically, basil compounds act as surface modifiers and hydrocolloidal stabilizers, limiting water and lipid loss and maintaining the structural integrity of the protein–water–fat network, thereby improving cooking yield and dimensional stability [20,72,73].

Overall, neither the addition of basil extract nor its microencapsulation significantly altered technological properties, in agreement with previous studies [74]. Burgers with added basil exhibited performance comparable to BHT-containing samples, indicating the potential of basil as a natural antioxidant alternative. Several authors have reported no effect of basil on centesimal composition [22,25] or on WHC and cooking losses in various meat products [75], including marinated goat jerky [76], marinated chicken [23] and chicken sausages [20]. Conversely, Albergamo et al. [77] observed nearly twofold higher cooking losses in control chicken nuggets compared with basil-enriched ones, likely due to the higher fibre content in the basil-containing formulations.

#### 3.4.2. pH and Objective Colour

**Figure 2:**
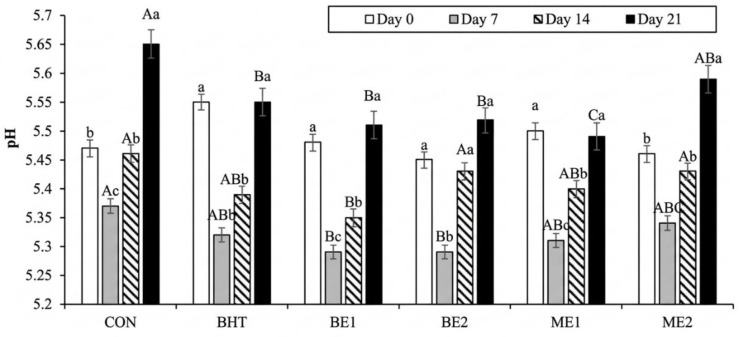
pH of burgers with different concentrations of basil extracts and microencapsulated basil extracts throughout storage time. CON—control. BHT—butylated hydroxytoluene 0.01%. BE1—addition of 0.01% basil extract. BE2—addition of 0.02% basil extract. ME1—addition of 0.01% microencapsulated basil extract. ME2—addition of 0.02% microencapsulated basil extract. Different uppercase letters indicate significant differences (*p* < 0.05) among treatments within a given day; different lowercase letters indicate significant differences (*p* < 0.05) among days within a given treatment.

Figure 2 shows the pH values between treatments over time. Significant differences in pH were observed between treatments and across storage times (*p* < 0.0001), with values ranging from 5.29 to 5.65, which are considered normal for beef burgers [78]. In all treatments, pH decreased from day 0 to day 7 and then increased from day 7 to day 21. No differences were observed between treatments at day 0 (*p* < 0.05); however, by day 21, all treatments except ME2 exhibited lower pH values than the CON. On day 21, BHT, BE1, BE2, and ME2 did not differ significantly, whereas ME1 showed the lowest pH. Overall, the addition of basil, whether encapsulated or not, effectively controlled pH changes during storage, similarly to BHT.

Most authors reported a decrease in pH when basil extract was added [25,70,76,79,80], which could be explained by the presence of the phenolic acids in the extract and by its antimicrobial activity [81]. Therefore, in the present study, it should be noted that the reduced antimicrobial activity of basil was insufficient to control bacterial growth by the end of the storage period.

The colour parameters L*, a*, b*, chroma (C*), and hue angle (H_ab_) are shown in Table 5. Overall, storage time exerted a greater influence on colour parameters than treatment. No differences between treatments were observed for any colour variable on days 14 or 21. On day 0, only b* was affected by treatment, with BE1 showing significantly lower values (*p* < 0.05) than CON and BHT. On day 7, only L* and C* were influenced by treatment. For L*, none of the treatments differed significantly from the CON (*p* > 0.05), although BE1 and ME2 exhibited lower values than BHT (*p* < 0.05). Regarding C*, BE1 and BE2 showed lower values than the CON (*p* < 0.05), while no basil-added treatment differed from BHT (*p* > 0.05).

A significant effect of storage time was observed for all colour variables except hue. Regardless of treatment, L* values increased from day 0 to day 7 and then decreased from day 7 to day 21, reflecting typical behaviour for fresh meat. Similarly, a*, b*, and C* values increased from day 0 to day 7. The BHT treatment maintained more stable colour over time, as indicated by the absence of significant changes in a*, b*, C*, and hue throughout storage.

The results showed that neither treatment nor storage time significantly affected hue values (*p* > 0.05). From a practical perspective, this indicates that all burgers exhibited comparable colouration, suggesting that colour is unlikely to influence sensory perception or consumer purchase intention.

Most authors reported that the inclusion of basil in meat products resulted in an increase in L* and b*, with no consensus on a* behaviour [22,25,70,79,80]. In a study conducted to measure the colour of basil leaves used in different marinades [76], it was concluded that, although basil presented yellowish pigments (b* = 18.39), which could partially explain the increase in b* when using basil, other plants present higher b* values, such as rosemary (b* = 22.54) or allium (b = 24.68). Accordingly, the impact of basil on colour can be considered moderate compared with that of other plants, which may be advantageous for consumer acceptance.

#### 3.4.3. Texture Profile Analysis (TPA)

The texture profile (Table 6) was analyzed only on days 0 and 21, as no major changes were expected due to the addition of basil extracts. Cohesiveness was the only parameter affected by treatment on both days 0 and 21 (*p* < 0.05). Storage time influenced chewiness in BHT and BE1, cohesiveness in the CON, and chewiness, elasticity, and cohesiveness in BE1, with all affected parameters showing an increase from day 0 to day 21. The results of the present study are consistent with most reports in the literature, which indicate that basil does not significantly affect meat textural parameters [22,25,76].

#### 3.4.4. Lipid Oxidation (Peroxide Index and TBARSs)

**Figure 3:**
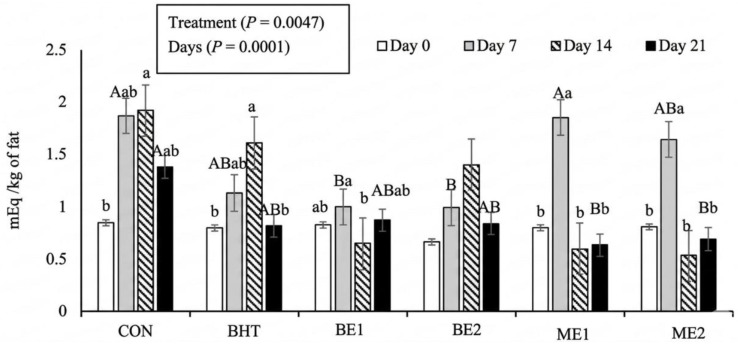
Peroxide index in burgers with different concentrations of basil extracts and microencapsulated basil extracts throughout storage time. CON—control. BHT—butylated hydroxytoluene 0.01%. BE1—addition of 0.01% basil extract. BE2—addition of 0.02% basil extract. ME1—addition of 0.01% microencapsulated basil extract. ME2—addition of 0.02% microencapsulated basil extract. Different uppercase letters indicate significant differences (*p* < 0.05) among treatments within a given day; different lowercase letters indicate significant differences (*p* < 0.05) among days within a given treatment.

Peroxide values (Figure 3) were influenced by both treatment (*p* = 0.0047) and storage time (*p* < 0.0001). No treatment effect was observed on days 0 and 14. On day 7, BE1 and BE2 exhibited lower values than the CON, whereas ME1 and ME2 did not differ significantly from the CON. Conversely, on day 21, BE1 and BE2 were similar to the CON, while ME1 and ME2 showed lower values than the CON. Across all storage times, none of the basil treatments differed from BHT.

Regarding storage time, peroxide values increased from day 0 to day 7 and then decreased, except for BE2, for which no significant effect of storage time was observed. The initial rise in peroxide values was more pronounced in CON, ME1, and ME2 than in BHT or BE treatments, suggesting that BHT, BE1, and BE2 were more effective at inhibiting peroxide formation during the early storage period.

The values obtained ranged from 0.66 to 1.92 mEq O_2_/kg of fat, substantially lower than the reference value of 20 mEq/kg of lipids, which is considered the maximum limit of peroxides for human food [82]. Few studies have looked at how adding basil affects the peroxide index. Boukour et al. [83] reported values around 15 mEq O_2_/kg of fat, which is clearly higher than the current results. These authors concluded that the effect of adding basil is inconsistent, since they reported no changes in pork fat but an increase in beef and sheep fat. This conclusion would be supported by the study by Abdulrahem, Al-Aubadi and Mohamed [20], who, working with chicken sausages in which the fat had been partially replaced by a gum with basil extract, reported that the inclusion of basil had no effect on the peroxide index.

**Figure 4:**
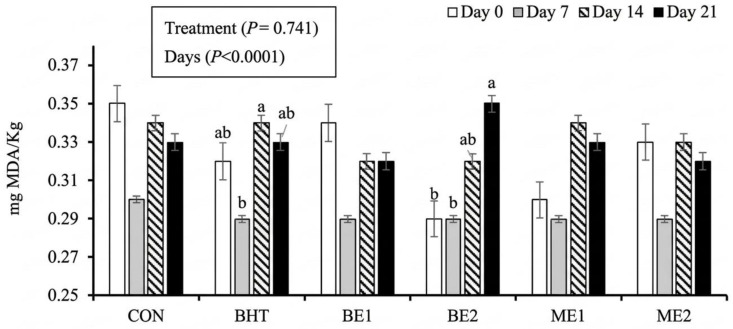
TBARS in burgers with different concentrations of basil extracts and microencapsulated basil extracts throughout storage time. CON—control. BHT—butylated hydroxytoluene 0.01%. BE1—addition of 0.01% basil extract. BE2—addition of 0.02% basil extract. ME1—addition of 0.01% microencapsulated basil extract. ME2—addition of 0.02% microencapsulated basil extract. Different lowercase letters indicate significant differences (*p* < 0.05) among days within a given treatment.

TBARS results are presented in Figure 4. Treatment had no significant effect on TBARS values (*p* = 0.741), whereas storage time exerted a significant effect (*p* < 0.0001). The effect of storage time was primarily observed in BHT and BE2, where values increased from day 7 onwards. These results are consistent with previous reports for meat products, in which TBARS values typically range from 0.2 to 1.2 mg MDA/kg [22,25,83] and remain below the maximum acceptable threshold of 2 mg MDA/kg, which is considered perceptible by consumers [84].

It has been reported by several authors that, for a given storage time, an increase in the concentration of basil extract is associated with a decrease in TBARS values [25], even though an increase in TBARS values over time occurred, as expected. On the contrary, other authors have stated no effect of basil extract on the TBARS values of several products, such as beef burgers [65] and linguiça [22], in agreement with the current results. Finally, several authors [79,85] have reported that, depending on the dose, plant additives can exert either protective or pro-oxidant effects, which may explain the observed increase in TBARS values in BE2 but not in BE1.

#### 3.4.5. Sensory Analysis

The sensory evaluation of the products was conducted using a liking test. Liking analysis assesses the degree of consumer acceptance based on individual ratings. In this study, the original scores were centred and scaled: centring removes differences in the average use of the scale among consumers, while scaling reduces variability in scale usage across individuals. The resulting standardized scores preserve the relative preference structure among products, allowing the final liking values to reflect relative acceptance while accounting for inter-individual differences in scoring.

**Figure 5:**
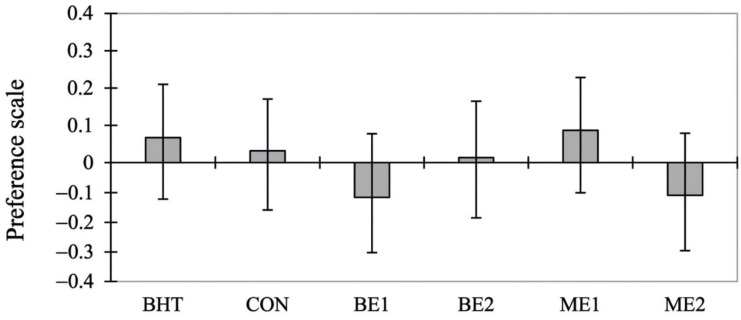
Preference values from a liking analysis for each product. CON—control. BHT—butylated hydroxytoluene 0.01%. BE1—addition of 0.01% basil extract. BE2—addition of 0.02% basil extract. ME1—addition of 0.01% microencapsulated basil extract. ME2—addition of 0.02% microencapsulated basil extract.

Figure 5 represents the Least Square Means for the preference liking of each product. It can be observed that the BE1 and ME2 treatments were less preferred than the others. Some studies have reported that increasing the concentration of basil extract enhances aroma and flavour without introducing off-flavours [65]. This may be attributed to the fact that basil is a familiar ingredient in many meat preparations, and thus its flavour is not perceived as unusual by consumers. Nevertheless, other studies [20,77] reported no effect of basil addition on the sensory profile of products and Macari, Stürza, Lung, Soran, Opriş, Balan, Ghendov-Moșanu, Cristian Vodnar and Cojocari [26] reported that sensory scores decreased when basil extract concentration increased. However, even after centering and scaling, the variability of the data remained too high to reveal clear patterns. Indeed, the ANOVA performed on these liking values was not significant (*p* = 0.639), indicating that the variables did not contribute meaningfully to the model.

A clustering analysis was subsequently conducted to determine whether consumer segmentation could reveal more discernible behavioural patterns. The analysis identified two clusters. Cluster 1 comprised 47 consumers, whereas Cluster 2 comprised 58 consumers. Means of liking values for each treatment and cluster, along with ANOVA *p*-values, are presented in Table 7. Significant differences between clusters were observed for BE1, ME1, and ME2, with higher values consistently recorded in cluster 2. Unfortunately, no supplementary data regarding psychographic variables of consumers (e.g., age, income, or consumption frequency) were available. The absence of this information limits the interpretability of the results, as it has been widely reported that lifestyle and psychographic factors can account for variability in consumer studies [86,87,88].

#### 3.4.6. Purchase Intention

Table 8 presents the relative percentages of valid responses for each product and category in the purchase intention test. Data are shown for the overall population (global) and for each cluster. A chi-square test was performed to assess differences between clusters. Some differences were observed between the global population and clustered data. Furthermore, significant differences in purchase intention were found between clusters for CON, BHT, and BE1 treatments (*p* = 0.021; values in Table 8 are highlighted in bold). Cluster 1 tended to prefer BHT and reject BE1, while cluster 2 showed a tendency to prefer BE2, although this was not statistically different from cluster 1. These results also indicate that liking scores and purchase intention do not necessarily correlate.

When consumers select a product in a supermarket, their decision is primarily based on external attributes, such as colour. During preparation and consumption, these expectations are either confirmed or contradicted, with prior experience with food in general and with the specific product playing a major role. Consequently, when a product is unfamiliar, extrinsic attributes have less influence, and purchase decisions are likely determined largely by individual consumer characteristics [87].

There is broad consensus in the literature that colour and fat content are the primary criteria used in meat selection, as they are strongly associated with perceptions of freshness and nutritional quality [89,90,91]. In the present study, fat content differed minimally among treatments. Regarding colour, differences were observed only for b*, with BE1 exhibiting lower values than CON and BHT. Future studies should collect additional consumer information, particularly regarding psychographic characteristics, to enable a clearer correlation between extrinsic attributes and sensory preferences.

## 4. Conclusions

Non-encapsulated basil extract exhibited higher total antioxidant capacity than its microencapsulated form and was rich in phenolic acids, flavonoids, aldehydes, isoflavones, proanthocyanidins, and flavonols, with naringenin as the predominant compound (294.72 mg/kg). It showed antimicrobial activity against Gram-positive Staphylococcus aureus (6.25 mg/mL) and Gram-negative Salmonella enteritidis, Escherichia coli, and Klebsiella pneumoniae (12.5 mg/mL). Incorporation of basil extract (0.01–0.02%) or its microencapsulated form can replace BHT in beef hamburgers, preserving physicochemical and technological properties and delaying lipid oxidation. Microencapsulation may reduce efficacy, warranting further investigation with alternative carriers or concentrations. Sensory acceptance was maintained at 0.01%, while flavour liking was reduced at 0.02%. Overall, the findings suggest that moderate levels of basil extract are technologically viable and sensorially acceptable for beef hamburgers.

## Figures and Tables

**Table 1:** The means and standard error for the antioxidant activity (DPPH and FRAP assays) of basil extract (BE), microencapsulated basil extract (ME), and BHT. Significance (*p*-values) for treatment effect.

	Antioxidant				
	BE	ME	BHT	s.e.	*p*-Value
DPPH (mg Trolox/g sample)	279.78 b	291.13 a	292.10 a	2.53	0.005
FRAP (µmol Fe^+2^/100 g sample)	6060.00 a	1884.00 b	605.00 c	1089.53	0.006

BE—basil extract; ME—microencapsulated basil extract; BHT—butylated hydroxytoluene; s.e.—standard error. a–c—different letters in the same row indicate a significant difference (*p* < 0.05) among compounds.

**Table 2:** The means and standard error for the phenolic compounds (mg/kg) in basil extract (BE) and microencapsulated basil extract (ME). Significance (*p*-values) for extract type.

Phenolic Compounds (mg/kg)	BE	ME	s.e.	*p*-Value
ACIDS				
Gallic acid	1.58	1.44	0.06	0.365
4-hydroxybenzoic acid	0.58	0.48	0.03	0.199
Vanillic acid	2.28	2.24	0.14	0.921
Syringic acid	2.82	2.66	0.17	0.729
trans-Cinnamic acid	11.97	12.92	0.94	0.708
Caftaric acid	4.25	1.97	0.70	0.062
Caffeic acid	21.71	17.47	2.40	0.490
p-coumaric acid	1.23	1.27	0.11	0.902
Ferulic acid	0.85	0.49	0.14	0.287
Fumaric acid	0.26	ND	0.07	<0.001
∑ phenolic acids	47.53	40.94		
STILBENES				
cis-Resveratrol	60.39	33.70	9.88	0.221
trans-Resveratrol	1.06	1.39	0.33	0.711
∑ stilbenes	61.45	35.09		
ALDEHYDES				
Vanillin	2.42	5.45	1.23	0.295
ISOFLAVONES				
Naringin	29.60	18.59	4.09	0.223
Hesperidin	21.98	12.82	3.42	0.228
Naringenin	294.72	233.10	50.56	0.648
Hesperitin	45.90	31.01	22.24	0.807
∑ isoflavones	392.19	295.51		
FLAVAN-3-OLS				
Catechin	0.26	0.19	0.02	0.186
Epicatehin gallate	2.35	2.24	0.12	0.765
Epigallocatechin gallate	2.11	1.53	0.17	0.026
∑ flavan-3-ols	4.72	3.96		
PROANTHOCYANIDINS				
Procyanidin A2	4.73	4.14	0.27	0.382
Procyanidin B1	0.45	0.38	0.02	0.166
Procyanidin B2	0.26	0.15	0.03	0.090
∑ proanthocyanidins	5.44	4.67		
FLAVONOLS				
Quercetin 3-glucoside	67.86	0.12	20.21	0.033
Rutin	0.36	54.48	15.64	0.001
Kaempferol 3-glucoside	11.46	8.38	1.18	0.248
Isorhamnetin	21.97	8.10	7.73	0.482
Quercetin hydrate	16.56	12.85	2.89	0.630
∑ Flavonols	118.20	83.92		
Total sum of phenolics	631.95	469.54		

BE—basil extract; ME—microencapsulated basil extract; ND—not detected; s.e.—standard error.

**Table 3:** Minimum inhibitory concentration and minimum bactericidal concentration of basil extract (BE) and microencapsulated basil extract (ME) against several strains.

	BE		ME	
	MIC (mg/mL)	MBC (mg/mL)	MIC (mg/mL)	MBC (mg/mL)
*Salmonella enteritidis* (ATCC 13076)	12.5	12.5	>12.5	>12.5
*Escherichia coli* (ATCC 10523)	12.5	12.5	>12.5	>12.5
*Escherichia coli* (ATCC 10536)	12.5	12.5	>12.5	>12.5
*Klebsiella pneumoniae* (ATCC 13883)	12.5	>12.5	>12.5	>12.5
*Staphylococcus aureus* (ATCC 33591)	6.25	6.25	>12.5	>12.5
*Staphylococcus aureus* (ATCC 25923)	6.25	6.25	>12.5	>12.5

MIC: minimum inhibitory concentration; MBC: minimum bactericidal concentration; BE: basil extract; ME: microencapsulated basil extract.

**Table 4:** Centesimal composition and technological properties of burgers with different concentrations of basil extract and microencapsulated basil extract.

	CON	BHT	BE1	BE2	ME1	ME2	s.e.	*p*-Values
Centesimal composition								
Moisture (%)	66.27 b	66.79 ab	69.87 a	67.75 ab	66.97 ab	66.80 ab	0.36	0.024
Ash (%)	2.45	2.47	2.46	2.33	2.52	2.55	0.02	0.124
Protein (%)	17.75	20.29	18.22	18.26	16.83	19.02	0.35	0.061
Fat (%)	14.01 a	13.26 ab	12.25 ab	11.65 b	12.30 ab	12.57 ab	0.21	0.012
Technological properties								
WHC (%)	15.87 ab	15.09 ab	16.65 a	15.67 ab	13.79 b	14.53 ab	0.51	0.039
Cooking loses (%)	24.80 a	16.25 b	18.03 ab	21.29 a	23.78 a	22.51 a	1.60	0.003
Diameter reduction (%)	21.96 a	21.28 a	19.70 a	15.74 b	19.90 a	18.66 ab	0.78	0.004

CON—control; BHT—butylated hydroxytoluene 0.01%; BE1—addition of 0.01% basil extract; BE2—addition of 0.02% basil extract; ME1—addition of 0.01% microencapsulated basil extract; ME2—addition of 0.02% microencapsulated basil extract; s.e.—standard error of the mean; a, b—different letters in the same row indicate a significant difference (*p* < 0.05) among treatments.

**Table 5:** Colour parameters of burgers with different concentrations of basil extracts and microencapsulated basil extracts throughout storage time.

	Treatments	0 Days	7 Days	14 Days	21 Days	s.e.	*p*-Value
L*	CON	41.15 b	51.63 aAB	43.65 b	40.26 b	3.31	<0.001
	BHT	39.51 b	52.22 aA	40.03 b	37.52 b	2.56	<0.001
	BE1	37.02 b	47.75 aB	40.52 b	37.98 b	3.15	0.002
	BE2	36.86 b	48.07 aA	40.39 b	39.56 b	3.80	0.003
	ME1	40.32 b	49.14 aAB	39.47 b	39.40 b	2.71	0.003
	ME2	38.61 b	46.61 aB	40.03 b	37.81 b	2.46	0.010
	s.e.	0.56	0.43	0.42	0.41		
	*p*-value	0.182	0.004	0.088	0.311		
a*	CON	12.68 b	15.38 a	13.32 ab	12.79 b	1.06	0.025
	BHT	13.06	15.39	13.92	14.27	1.53	0.143
	BE1	11.55 b	16.40 a	14.37 ab	13.94 ab	0.89	0.007
	BE2	13.07 b	17.33 a	14.48 b	13.11 b	2.16	0.001
	ME1	12.03 b	15.58 a	14.82 a	13.76 ab	2.12	0.009
	ME2	12.05 b	15.44 a	14.42 ab	13.45 ab	1.31	0.035
	s.e.	0.72	0.28	0.23	0.23		
	*p*-value	0.587	0.281	0.549	0.481		
b*	CON	12.41 A	14.34	13.78	12.71	1.29	0.0616
	BHT	13.00 A	15.06	12.92	13.25	1.29	0.0663
	BE1	10.38 cB	14.71 a	12.88 b	12.72 b	0.75	<0.001
	BE2	11.37 bAB	15.46 a	13.02 b	13.47 ab	2.22	0.001
	ME1	12.20 bAB	14.87 a	13.32 ab	13.23 ab	1.92	0.045
	ME2	11.92 bAB	14.67 a	13.52 ab	12.98 ab	1.08	0.045
	s.e.	0.22	0.21	0.20	0.15		
	*p*-value	0.037	0.747	0.742	0.633		
C*	CON	17.74 b	20.93 aB	19.32 ab	18.25 ab	1.66	0.039
	BHT	18.06 b	21.63 aAB	19.52 a	19.49 a	1.97	0.051
	BE1	15.78 b	22.18 aA	19.52 a	19.09 a	0.99	<0.001
	BE2	17.51 b	25.31 aA	19.78 a	19.16 a	2.40	<0.001
	ME1	17.28 a	21.45 abAB	20.04 ab	19.34 b	2.78	0.030
	ME2	17.34 b	21.44 aAB	19.72 ab	18.71 ab	1.64	0.018
	s.e.	0.31	0.40	0.27	0.22		
	*p*-value	0.388	0.046	0.979	0.626		
Hue	CON	45.38	44.81	44.88	43.90	0.78	0.923
	BHT	45.22	45.61	47.05	46.63	0.47	0.488
	BE1	48.98	46.73	47.66	47.98	0.80	0.800
	BE2	50.06	46.82	47.84	44.55	0.83	0.161
	ME1	45.16	45.69	47.53	45.60	0.56	0.47
	ME2	46.27	46.08	47.54	46.31	0.64	0.841
	s.e.	0.65	0.56	0.41	0.41		
	*p*-value	0.150	0.906	0.319	0.090		

CON—control; BHT—butylated hydroxytoluene 0.01%; BE1—addition of 0.01% basil extract; BE2—addition of 0.02% basil extract; ME1—addition of 0.01% microencapsulated basil extract; ME2—addition of 0.02% microencapsulated basil extract; s.e.—standard error of the mean. Different uppercase letters indicate significant differences (*p* < 0.05) among treatments within a given day; different lowercase letters indicate significant differences (*p* < 0.05) among days within a given treatment.

**Table 6:** Texture profile of burgers with different concentrations of basil extract and microencapsulated basil extract during storage for 21 days.

Variables	Treatments	0 Days	21 Days	s.e.	*p*-Value
Hardness (N)	CON	21.10	14.71	2.034	0.191
	BHT	14.49	24.02	2.211	0.120
	BE1	14.13	24.01	2.244	0.159
	BE2	11.89	15.61	2.896	0.566
	ME1	16.24	18.90	3.289	0.706
	ME2	15.97	19.59	3.688	0.650
	s.e.	1.422	2.055		
	*p*-value	0.547	0.708		
Chewiness (N/mm)	CON	14.52	13.42	1.376	0.71
	BHT	9.78	19.32	1.396	0.027
	BE1	8.29	19.35	1.086	0.007
	BE2	6.80	10.16	1.202	0.234
	ME1	11.28	14.39	2.506	0.568
	ME2	10.81	13.45	2.475	0.623
	s.e.	0.923	1.116		
	*p*-value	0.295	0.193		
Elasticity (mm)	CON	0.91	1.09	0.051	0.145
	BHT	0.91	1.00	0.022	0.098
	BE1	0.83	1.00	0.008	<0.001
	BE2	0.82	0.90	0.029	0.242
	ME1	0.91	1.00	0.022	0.125
	ME2	0.89	0.91	0.038	0.804
	s.e.	0.018	0.018		
	*p*-value	0.532	0.093		
Cohesiveness	CON	0.76 A	0.84 A	0.007	0.006
	BHT	0.74 AB	0.79 B	0.009	0.057
	BE1	0.70 B	0.79 B	0.006	0.002
	BE2	0.71 B	0.74 C	0.007	0.067
	ME1	0.74 AB	0.76 B	0.008	0.347
	ME2	0.75 AB	0.76 B	0.007	0.643
	s.e.	0.005	0.004		
	*p*-value	0.009	<0.001		

CON—control; BHT—butylated hydroxytoluene 0.01%; BE1—addition of 0.01% basil extract; BE2—addition of 0.02% basil extract; ME1—addition of 0.01% microencapsulated basil extract; ME2—addition of 0.02% microencapsulated basil extract; s.e.—standard error of the mean. Different uppercase letters indicate significant differences (*p* < 0.05) among treatments within a given day.

**Table 7:** Least square means and *p*-values for liking scores for each product in each cluster. *p*-values for ANOVA procedure with cluster as the main effect.

	CON	BHT	BE1	BE2	ME1	ME2
Cluster 1 (n = 47)	6.9	7.0	6.0 y	6.8	6.2 y	7.2 y
Cluster 2 (n = 58)	6.5	6.5	7.0 x	6.6	7.1 x	6.2 x
*p*-value	0.183	0.097	0.001	0.415	0.001	0.003

CON—control. BHT—butylated hydroxytoluene 0.01%. BE1—addition of 0.01% basil extract. BE2—addition of 0.02% basil extract. ME1—addition of 0.01% microencapsulated basil extract. ME2—addition of 0.02% microencapsulated basil extract. x, y.—different letters indicate a significant difference (*p* < 0.05) among clusters.

**Table 8:** Purchase intention. Relative percentages of valid answers in each category, for each product and cluster. Percentages in bold are those that differed significantly between clusters (*p* < 0.05).

Treatment		I Would Certainly Not Buy It.	I Probably Wouldn’t Buy It.	Maybe Yes/Maybe No	I Probably Would Buy It.	I Would Certainly Purchase It.
CON	Global	8.4	20.6	23.4	23.4	24.3
	Cluster 1	12.2	**10.2**	**34.7**	20.4	22.4
	Cluster 2	5.2	**29.3**	**13.8**	25.9	25.9
BHT	Global	3.7	14.0	22.4	39.3	20.6
	Cluster 1	2.0	8.2	20.4	38.8	**30.6**
	Cluster 2	5.2	19.0	24.1	39.7	**12.1**
BE1	Global	7.5	17.8	27.1	27.1	20.6
	Cluster 1	**14.3**	24.5	22.4	26.5	12.2
	Cluster 2	**1.7**	12.1	31.0	27.6	27.6
BE2	Global	3.7	15.9	28.0	3.4	15.0
	Cluster 1	4.1	14.3	34.7	28.6	18.4
	Cluster 2	3.4	17.2	22.4	44.8	12.1
ME1	Global	5.6	14.0	28.0	29.9	22.4
	Cluster 1	10.2	14.3	32.7	26.5	16.3
	Cluster 2	1.7	13.8	24.1	32.8	27.6
ME2	Global	10.3	14.0	21.5	28.0	26.2
	Cluster 1	6.1	8.2	22.4	30.6	32.7
	Cluster 2	13.8	19.0	20.7	25.9	20.7

CON—control. BHT—butylated hydroxytoluene 0.01%. BE1—addition of 0.01% basil extract. BE2—addition of 0.02% basil extract. ME1—addition of 0.01%. ME2—addition of 0.02% microencapsulated basil extract.

## Data Availability

The data that support the findings of this study are available from the corresponding author upon reasonable request.
